# Circulating adiponectin levels are lower in Latino versus non-Latino white patients at risk for cardiovascular disease, independent of adiposity measures

**DOI:** 10.1186/1472-6823-11-13

**Published:** 2011-07-07

**Authors:** Rocio I Pereira, Cecilia CL Wang, Patrick Hosokawa, L Miriam Dickinson, Michel Chonchol, Mori J Krantz, John F Steiner, Daniel H Bessesen, Edward P Havranek, Carlin S Long

**Affiliations:** 1 Denver Health Medical Center, 660 Bannock Street, Denver, CO 80204, USA; 2 University of Colorado Denver, Anschutz Medical Campus, P.O. Box 6511, MS8106, Aurora, CO 80045, USA; 3 Denver Veterans Affairs Medical Center, 1055 Clermont Street, Denver, CO 80220, USA; 4 Institute for Health Research, Kaiser Permanente, P.O. Box 378066, Denver, CO 80237, USA

## Abstract

**Background:**

Latinos in the United States have a higher prevalence of type 2 diabetes than non-Latino whites, even after controlling for adiposity. Decreased adiponectin is associated with insulin resistance and predicts T2DM, and therefore may mediate this ethnic difference. We compared total and high-molecular-weight (HMW) adiponectin in Latino versus white individuals, identified factors associated with adiponectin in each ethnic group, and measured the contribution of adiponectin to ethnic differences in insulin resistance.

**Methods:**

We utilized cross-sectional data from subjects in the Latinos Using Cardio Health Actions to reduce Risk study. Participants were Latino (n = 119) and non-Latino white (n = 60) men and women with hypertension and at least one other risk factor for CVD (age 61 ± 10 yrs, 49% with T2DM), seen at an integrated community health and hospital system in Denver, Colorado. Total and HMW adiponectin was measured by RIA and ELISA respectively. Fasting glucose and insulin were used to calculate the homeostasis model insulin resistance index (HOMA-IR). Variables independently associated with adiponectin levels were identified by linear regression analyses. Adiponectin's contribution to ethnic differences in insulin resistance was assessed in multivariate linear regression models of Latino ethnicity, with logHOMA-IR as a dependent variable, adjusting for possible confounders including age, gender, adiposity, and renal function.

**Results:**

Mean adiponectin levels were lower in Latino than white patients (beta estimates: -4.5 (-6.4, -2.5), p < 0.001 and -1.6 (-2.7, -0.5), p < 0.005 for total and HMW adiponectin), independent of age, gender, BMI/waist circumference, thiazolidinedione use, diabetes status, and renal function. An expected negative association between adiponectin and waist circumference was seen among women and non-Latino white men, but no relationship between these two variables was observed among Latino men. Ethnic differences in logHOMA-IR were no longer observed after controlling for adiponectin levels.

**Conclusions:**

Among patients with CVD risk, total and HMW adiponectin is lower in Latinos, independent of adiposity and other known regulators of adiponectin. Ethnic differences in adiponectin regulation may exist and future research in this area is warranted. Adiponectin levels accounted for the observed variability in insulin resistance, suggesting a contribution of decreased adiponectin to insulin resistance in Latino populations.

## Background

Latinos in the United States have a higher incidence and prevalence of insulin resistance, and consequently type 2 diabetes mellitus [1,2], compared to non-Latino whites. Though this observation has been attributed, at least in part, to a higher rate of obesity in Latinos [1], insulin resistance and type 2 diabetes are more prevalent in Latinos compared to whites even after controlling for weight differences [1,3-5].

Adiponectin, a circulating protein made primarily by adipose tissue [6-9], has been identified as a mediator of whole body insulin sensitivity [10-12]. Adiponectin levels are positively correlated with insulin sensitivity [13], and decreased levels of adiponectin are observed in insulin-resistant conditions [13,14] including type 2 diabetes mellitus. Adiponectin administration in animal models [12], and therapeutic interventions that increase adiponectin in humans such as anti-diabetic agents in the thiazolidinedione (TZD) class, are associated with improved insulin sensitivity [15,16]. Furthermore, decreased adiponectin levels precede decreases in whole body insulin sensitivity in humans [14], supporting a role for adiponectin as a mediator of insulin sensitivity. Though much research has been done on adiponectin, regulation of adiponectin levels remains poorly understood. Adiponectin levels have been predominantly associated with measures of adiposity [13,17] but are also associated with age [18], gender [19], presence of diabetes [20], renal function [21] and TZD use [22]. Associations with other factors including use of angiotensin-converting enzyme inhibitors (ACEI) or angiotensin receptor blockers (ARB) [23], acetylsalicylic acid [24], and statins [25] remain controversial.

Racial minorities (Black, American Indian, and Asian) have been reported to have lower circulating adiponectin levels compared to white individuals [5,26-28]. However, we are aware of only one previous report of decreased adiponectin levels in Latinos compared to non-Latino whites [5], and studies identifying determinants of adiponectin levels in Latinos or exploring the contribution of decreased adiponectin to decreased insulin sensitivity in this population are lacking. Furthermore, no information is currently available on ethnic differences in high-molecular-weight (HMW) adiponectin, which is thought to be the active form of the hormone.

In the present analysis we compared total and HMW adiponectin levels in a heterogeneous population of Latino (mostly Mexican-American) and non-Latino white patients with chronic hypertension, and measured the independent association between adiponectin levels and ethnicity. We identified determinants of adiponectin levels and explored the relationship between adiponectin and adiposity (BMI and waist circumference) in each ethnic group and in the population as a whole. Lastly, we assessed the contribution of decreased adiponectin levels to ethnic differences in HOMA-IR (a surrogate marker of insulin resistance) independent of other known predictors of insulin resistance.

## Methods

### Participants

Subjects in the Latinos Using Cardio Health Actions to Reduce Risk (LUCHAR) study were included in this cross-sectional study. The overall aim of LUCHAR is to improve cardiovascular disease (CVD) prevention in Latinos, taking into account a higher prevalence of cardiovascular risk factors, lower treatment rates, and unique social, cultural, language, and access-to-care barriers when compared to non-Latino white patients. The LUCHAR cohort included in the present study was recruited to investigate surrogate markers of CVD in Latinos. Laboratory measures specific to the present study were measured from stored blood samples obtained from the LUCHAR project. The study protocol was approved by the Colorado Multiple Institutional Review Board (COMIRB) and written consent was obtained from all participants. All study procedures were conducted according to the principles expressed in the Declaration of Helsinki. The study was conducted at Denver Health Medical Center (DHMC), an integrated community health and safety-net hospital system in Denver, Colorado, United States of America. Potential subjects were identified using the DHMC electronic hypertension registry comprised of patients with at least one diagnostic (ICD-9) code for hypertension, seen in the DHMC system between July 1, 2002 and June 30, 2007. Health information, including past medical history and medication use, were obtained from an initial chart review and from patient self-report. Patients were eligible for the study if they had a prior diagnosis of hypertension, were ≥18 years of age, attended a clinic in the DHMC system, and were of either self-reported Latino or non-Latino white race/ethnicity. Subjects were also required to have self-reported history or diagnosis of at least one other cardiovascular risk factor including diabetes, dyslipidemia, obesity, chronic kidney disease/microalbuminuria, current smoking, or older age (men >55 years, women >65 years). Patients were excluded if they had a known history of cardiovascular disease (myocardial infarction or coronary revascularization), history of stroke, cerebrovascular revascularization, peripheral arterial disease, chronic heart failure, valvular heart disease, end-stage renal disease, inflammatory disease or vasculitis. Eligible participants were contacted via telephone and invited to schedule a study visit. All individuals meeting eligibility criteria and consenting to participate in the study were included in the LUCHAR cohort. Subject recruitment was continued until the predetermined number of participants needed for the analyses of CVD outcomes was achieved. All 179 subjects in the final LUCHAR cohort were included in the present analysis.

### Protocol

Eligible participants were contacted via telephone and scheduled for a study visit. Participants were asked to fast for the 8 hours prior to their study visit. At the study visit, subjects completed a personal history questionnaire and reviewed a list of current medications. Height, weight, waist circumference, and blood pressure were measured. Fasting blood samples were drawn for basic chemistry panel, fasting lipids, complete blood count, HbA_1c_, and high sensitivity C-reactive protein (hsCRP). Insulin, total adiponectin, and HMW adiponectin were measured from stored plasma samples from this study visit.

### Laboratory Assays

Insulin and total adiponectin were measured by RIA (Diagnostic Systems Laboratory, Inc., Webster, TX; and LINCO Research, Inc.; St. Charles, MO respectively). Intra-assay precision for the total adiponectin assay is 1.78-3.59% CV, and inter-assay precision is 6.90-9.25% CV. HMW adiponectin was measured by ELISA (Millipore, Billerica, MA); intra-assay precision is 2.5-4.7% CV and inter-assay precision is 5.8-6.9% CV. All other labs were measured by the Denver Health Core Laboratory using standard procedures.

### Calculations

The homeostasis model insulin resistance index [29] was calculated from fasting insulin and glucose levels using the formula: HOMA-IR = (fasting insulin in mU/l × (fasting plasma glucose in mg/dl)/18))/22.5. Body mass index (BMI) was calculated as weight divided by squared height. Estimated GFR (eGFR) was calculated using the 4-variable abbreviated MDRD formula [30]: eGFR = 186 × (serum creatinine)^-1.154 ^× (age)^-0.203 ^× (0.742 if female) × (1.210 if black).

### Statistical Analyses

SAS version 9.2 (SAS Institute, Inc., Cary, NC) was used for all statistical analyses. Means, medians, and standard deviations were generated for each variable, stratified by gender and ethnicity. Group differences were assessed by Students t-test. Wilcoxon's test was used for non-normally distributed variables. A two-sided p value < 0.05 was considered statistically significant. Kendall's Tau bivariate analyses were performed between total or HMW adiponectin and variables thought likely to predict adiponectin level, both within each ethnic group and in the entire population. In addition to known associates of adiponectin levels including age, gender, BMI, waist circumference, diagnosis of diabetes, renal function, and use of TZD, we included use of medications associated with adiponectin in other populations (ACEI and ARB, acetylsalicylic acid, and statins) as exploratory variables. The correlation between total adiponectin and HMW adiponectin was also measured in order to verify an expected association between these two variables.

To identify independent determinants of adiponectin, multivariate linear regression analyses were performed with total or HMW adiponectin as the dependent variable. An interaction term for ethnicity x waist circumference was used to assess a possible moderating effect of waist circumference on the relationship between ethnicity and adiponectin.

We measured adiponectin's contribution to the observed ethnic difference in insulin resistance by performing multivariate regression analyses with logarithmically transformed HOMA-IR as the outcome variable. Initially, ethnicity, confounders (age, gender, TZD use, Statin use, estimated GFR, and diabetes status), and either BMI or waist circumference were entered into the model. Next, total adiponectin was added to each model. Finally, HMW adiponectin was substituted for total adiponectin.

## Results

### Subject characteristics

Clinical and biochemical characteristics are shown in Table 1, stratified by ethnicity and gender. Our subject cohort was made up of 119 Latinos (81 women, 38 men) and 60 non-Latino whites (39 women, 21 men). Though individuals from any Latino sub-group were eligible to participate, >97% of participants in the Latino cohort were of Mexican-American descent, reflective of the make-up of the Latino population in the Denver area. Average age was 61.4 +/- 9.7 years and mean BMI was 33.1 +/- 6.7 kg/m^2^. Overall, 49% of subjects had type 2 diabetes. There were no ethnic group differences in BMI, but mean waist circumference was lower in the Latina women compared to the white women. White women had lower mean estimated GFR than Latina women, but mean eGFR was within the normal range for all groups. In regards to medication use, TZD use was higher and ASA use was lower in Latino men compared to white men, but use of ACEI/ARB and statin was not different in the two ethnic groups. No ethnic group differences were observed in other demographic or clinical characteristics (age, gender representation, fasting lipids, systolic blood pressure, or hs-CRP).

**Table 1:** Clinical and biochemical characteristics of the study subjects (n = 179)

Variable	Latino (n = 119)		Non-Latino white (n = 60)	
	Men (n = 38)	Women (n = 81)	Men (n = 21)	Women (n = 39)
Age, yr	60.2 ± 10.2	60.6 ± 10.4	61.7 ± 9.6	63.9 ± 7.5
BMI, kg/m^2^	32.6 ± 5.4	33.0 ± 6.3	31.3 ± 8.0	34.7 ± 7.6
Waist Circumference, cm	109.9 ± 13.7 ^**a**^	102.5 ± 15.4	112.0 ± 20.3	109.0 ± 16.0 ^**c**^
Type 2 Diabetes	57.9%	51.9%	42.9%	35.9%
HOMA-IR	5.3 (3.4-9.2)	4.5 (3.0-6.5)	4.1 (2.0-6.9)	3.7 (2.3-5.6)
Log(HOMA-IR)	1.7 ± 0.7	1.4 ± 0.7	1.2 ± 1.2	1.2 ± 0.9
Fasting Insulin, μU/ml	18 (12-26)	17 (12-23)	16 (8-25)	16 (9-22)
Estimated GFR, ml/min	89.6 ± 30.7	90.7 ± 24.4	83.5 ± 22.0	74.9 ± 23.8 ^**d**^
Total Cholesterol, mg/dl	171 ± 46.2	181 ± 36.6	182 ± 41.3	192 ± 41.3
Triglycerides, mg/dl	162 (104-241)	159 (120-204)	135 (91-164)	146 (108-207)
LDL, mg/dl	91.9 ± 35.6	95.3 ± 32.7	99.8 ± 32.8	103.3 ± 31.8
HDL, mg/dl	43.4 ± 9.3 ^**b**^	53.0 ± 12.5	48.0 ± 12.4	55.1 ± 13.9
Systolic BP, mm Hg	141 ± 19.4	144 ± 23.1	141 ± 24.1	139 ± 21.7
hs-CRP, mg/L	2.8 (1.0-5.3)	3.5 (1.7-6.5)	1.8 (0.9-3.7)	3.5 (1.2-8.5)
ACEI or ARB use	68.4%	56.8%	42.9%	53.9%
TZD use	18.4%	14.8%	0% ^**c**^	7.7%
ASA use	26.3%	30.9%	57.1% ^**c**^	35.9%
Statin use	63.2%	43.2%	52.4%	56.40%
Total Adiponectin, μg/ml	4.6 (3.8-6.5) ^**b**^	7.0 (4.9-11.4)	6.0 (5.0-10.6) ^**ac**^	11.4 (7.3-17.1) ^**d**^
HMW Adiponectin*, μg/ml	2.3 (1.2-3.7) ^**a**^	2.8 (1.7-5.0)	2.9 (2.0-3.9) ^**a**^	4.4 (2.8-7.9) ^**d**^

Results are Mean ± sd, Median (interquartile range), or % ^a ^P < 0.05, ^b ^P < 0.01 for comparison by sex within ethnic group ^c ^P < 0.05, ^d ^P < 0.01 for comparison by ethnic group within sex

* HMW adiponectin was available on 36 Latino men, 71 Latino women, 20 white men, 39 white women.

BMI, body mass index; HOMA-IR, homeostasis model assessment insulin resistance index; GFR, glomerular filtration rate; LDL, low density lipoprotein; HDL, high density lipoprotein, hs-CRP, high-sensitivity C reactive protein; ACEI, angiotensin converting enzyme inhibitor; ARB, angiotensin receptor blocker; TZD, thiazolidinedione; HMW, high-molecular-weight.

### Decreased total and HMW adiponectin in Latinos

**Figure 1:**
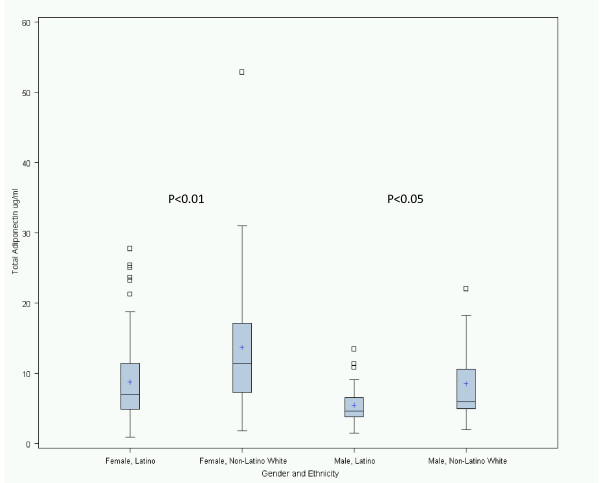
**Total adiponectin concentrations in Latinos versus non-Latino white patients, by gender**. Boxes represent median (*line *in the middle of the boxes), mean (*+ symbol *inside boxes), and interquartile ranges (25^th ^and 75^th ^percentile; *top *and *bottom *of boxes). The *error bars *are drawn to the values 1.5 times the interquartile range (IQR) below the 25^th ^percentile and 1.5 × IQR above the 75^th ^percentile.

Median total and HMW adiponectin was lower in Latinos compared to non-Latino whites (Table 1 and figure 1). Total adiponectin in Latinos and non-Latinos was 7.0 (4.9-11.4) versus 11.4 (7.3-17.1) μg/ml in women, p = 0.001 and 4.6 (3.8-6.5) versus 6.0 (5.0-10.6) μg/ml in men, p = 0.02 (figure 1). Similarly, HMW adiponectin was lower in the Latino versus non-Latino group (2.8 (1.7-5.0) versus 4.4 (2.8-7.9) μg/ml in women, p = 0.009 and 2.3 (1.2-3.7) versus 2.9 (2.0-3.9) μg/ml in men, p = 0.1). HMW/total adiponectin ratio was not different between the two groups.

### Correlates of adiponectin

In order to identify additional correlates of adiponectin levels, bivariate associations between total or HMW adiponectin and variables thought likely to influence adiponectin levels were assessed (Table 2). Strength and magnitude of correlations with total and HMW adiponectin were similar, and an expected strong association between total and HMW adiponectin was observed. Older age, and female gender, were positively associated with adiponectin levels in both ethnic groups. TZD use was significantly associated with adiponectin in the Latino group only. ACEI/ARB, aspirin, and Statin use was not associated with adiponectin levels in the population as a whole. Adiponectin levels were not correlated to diabetes status or estimated GFR. Though adiponectin levels were not correlated with BMI in either ethnic group or in the population as a whole in the bivariate analyses, we observed a negative association between adiponectin levels and waist circumference in the non-Latino group but not in the Latino group.

**Table 2:** Kendall's Tau correlations with total and HMW adiponectin

Variable	Total Adiponectin			HMW Adiponectin		
	**Latino**	**Non-Latino**	**All subjects**	**Latino**	**Non-Latino**	**All subjects**

Age in years	0.21^c^	0.19^a^	0.21^d^	0.22^c^	0.17	0.22^d^
Female Gender	0.25^c^	0.27^a^	0.24^d^	0.16^a^	0.23^a^	0.17^b^
BMI	-0.02	-0.09	-0.04	-0.06	-0.12	-0.08
Waist Circ.	-0.03	-0.24^b^	-0.07	-0.05	-0.22^a^	-0.08
Diabetes	0.05	0.07	-0.03	0.05	-0.13	-0.05
Estimated GFR	-0.10	0.03	-0.09	-0.12	0.07	-0.09
ACEI or ARB	-0.07	0.10	-0.03	-0.07	0.10	-0.03
TZD	0.23^b^	0.07	0.15^a^	0.25^b^	0.16	0.19^b^
ASA	0.03	-0.21^a^	-0.02	0.07	-0.05	0.06
Statin	-0.17^a^	-0.07	-0.11	-0.13	0.03	-0.05
HMW Adiponectin	0.68^d^	0.62^d^	0.66^d^	-	-	-

^a ^p ≤ 0.05

^b ^p < 0.01

^c ^p < 0.001

^d ^p < 0.0001

HMW, high-molecular-weight; BMI, body mass index; GFR, glomerular filtration rate; ACEI, angiotensin converting enzyme inhibitor; ARB, angiotensin receptor blocker; TZD, thiazolidinedione; ASA, acetylsalicylic acid.

### Adiponectin is negatively associated with waist circumference in non-Latino white men but not in Latino men

**Figure 2:**
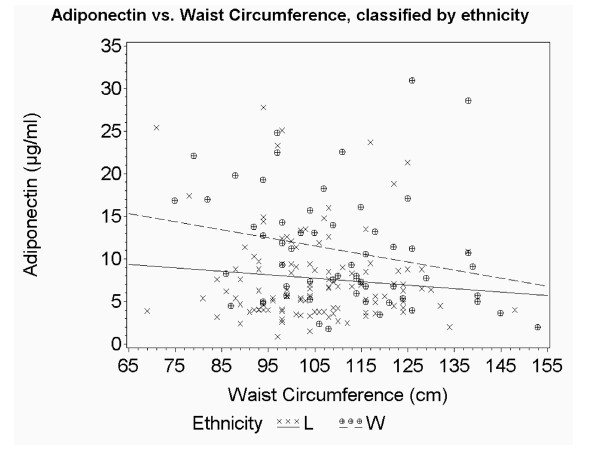
**Relationship between total adiponectin level and waist circumference in Latino and non-Latino white patients**. Scatter-plot and predicted regression lines showing the relationship between total adiponectin and waist circumference by ethnicity. Data for Latino patients is represented by *x symbols *and *solid line; *for non-Latino patients by *circular symbols *and *dashed line*.

**Figure 3:**
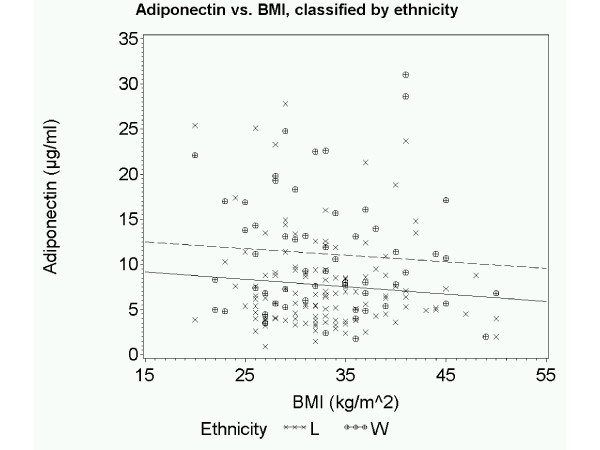
**Relationship between total adiponectin level and BMI in Latino and non-Latino white patients**. Scatter-plot and predicted regression lines showing the relationship between total adiponectin and BMI by ethnicity. Data for Latino patients is represented by *x symbols *and *solid line; *for non-Latino patients by *circular symbols *and *dashed line*.

To further explore our observation of an association between waist circumference and adiponectin in the white participants but not the Latino group, we used an interaction term for ethnicity x waist circumference. As demonstrated by the plotted regression lines of waist circumference versus adiponectin by ethnicity (Figure 2) we found that waist circumference was negatively associated with adiponectin in the non-Latino group, whereas no association was found between these two variables in the Latino group (p value for interaction <0.05). When waist circumference and adiponectin values are plotted by ethnicity and gender (Additional file 1: Figure S1), it becomes apparent that the observed ethnic difference in the waist circumference and adiponectin association is driven by the men, with Latino men having low adiponectin regardless of waist circumference. Thus, at any given waist circumference, adiponectin is lower in Latinos than in non-Latino whites. The only possible exception to this observation is at the higher waist circumference measures where the regression lines for the men cross and men in both ethnic groups have low adiponectin. The relationship between adiponectin and BMI (Figure 3; Additional File 2: Figure S2) is similar, with lower adiponectin in Latinos versus non-Latino whites at any given BMI.

### Latino ethnicity is negatively associated with decreased adiponectin

In the multivariate analysis examining the relationship between ethnicity and adiponectin levels (Table 3 and Additional File 3: Table S1), Latino ethnicity was the strongest negative predictor of both total (-4.7; 95% CI -6.7, -2.8; p < 0001) and HMW (-1.8; 95% CI -2.9, -0.8; p = 0.001) adiponectin, independent of all other variables included in the model (age, gender, TZD use, Statin use, estimated GFR, diabetes status, and waist circumference/BMI).

**Table 3:** Linear regression analysis: Association between adiponectin and clinical characteristics

Dependent variable	Estimate (95% CI)	*P*
**Total adiponectin**		
Intercept	9.4 (-0.5, 19.3)	
Latino	-4.5 (-6.4, -2.5)	<0.0001
Age (per 5 years)	0.7 (0.2, 1.2)	0.0041
Female Gender	3.7 (1.8, 5.6)	0.0027
TZD use	5.1 (2.2, 8.0)	0.0001
BMI (per 5 kg/m^2^)	-1.0 (-1.5,-0.2)	0.0054
Statin use	-2.4 (-4.3,-0.6)	0.0104
Estimated GFR (per ml/min)	-0.02 (-0.05, 0.02)	0.41
Diabetes	-0.3 (-2.3, 1.7)	0.76
**HMW adiponectin**		
Intercept	4.4 (-1.0, 9.9)	
Latino	-1.6 (-2.7, -0.5)	0.0047
Age (per 5 years)	0.3 (0.1, 0.6)	0.0122
Female Gender	1.4 (0.3, 2.4)	0.0107
TZD use	2.9 (1.3, 4.5)	0.0007
BMI (per 5 kg/m^2^)	-0.5 (-1.0,-0.15)	0.0083
Statin use	-0.9 (-1.9, 0.2)	0.1007
Estimated GFR (per ml/min)	-0.008 (-0.03, 0.01)	0.4176
Diabetes	-0.5 (-1.6, 0.6)	0.4029

Estimates reflect changes from group mean for total or HMW adiponectin (μg/ml) (as reflected by the intercept of the regression model) for each predictive factor.

TZD, thiazolidinedione; BMI, body mass index; GFR, glomerular filtration rate; HMW, high-molecular-weight.

### Adiponectin accounts for the observed ethnic-specific variability in insulin resistance

In order to measure the contribution of adiponectin levels to ethnic-group differences in insulin resistance, we performed multivariate linear regression analyses in the entire cohort, with logHOMA-IR as the outcome variable (table 4 and Additional File 3: Table S2). Though Latino ethnicity showed a strong positive association with logHOMA-IR when ethnicity and confounders (age, gender, TZD use, statin use, estimated GFR, waist circumference/BMI) were included in the model (Model 1), with the addition of total adiponectin level (Model 2), the association with Latino ethnicity was no longer significant. Similarly, including HMW instead of total adiponectin and adjusting for confounders and ethnicity (Model 3), HMW adiponectin level was inversely associated with logHOMA-IR, and the association between Latino ethnicity and logHOMA-IR was no longer significant.

**Table 4:** Multivariate linear regression analysis: Association between logHOMA-IR and BMI, ethnicity, and adiponectin

Models (outcome = logHOMA-IR)	Estimate (95% CI)	*P*
Model 1: R^2 ^= 0.18		
Latino	0.34 (0.095, 0.59)	0.0068
Model 2: R^2 ^= 0.24		
Latino	0.19 (-0.56, 0.44)	0.1267
Total Adiponectin	-0.035 (-0.054, -0.017)	0.0002
Model 3: R^2 ^= 0.27		
Latino	0.18 (-0.061, 0.42)	0.1421
HMW Adiponectin	-0.092 (-0.13, -0.056)	<0.0001

All models controlled for BMI, age, gender, statin use, and eGFR.

logHOMA-IR, log of homeostasis model assessment insulin resistance index; BMI, body mass index; HMW, high-molecular-weight; eGFR, estimated glomerular filtration rate.

## Discussion

In the present study, we found decreased total and HMW adiponectin levels in Latino compared to non-Latino white patients with CVD risk. Lower adiponectin levels in the Latino group were independent of BMI and other factors known to affect adiponectin, and seemed to account for the increased insulin resistance observed in this group compared to non-Latino whites. Ethnic group differences in HMW adiponectin paralleled those observed in total adiponectin, and there were no differences in HMW/total adiponectin ratios in the two ethnic groups. We identified correlates of total and HMW adiponectin levels in the two ethnic groups, confirming associations of adiponectin levels with age, gender, adiposity, and thiazolidinedione use previously reported in majority populations. Though there was no association between BMI and adiponectin on univariate analyses, the expected negative association between these two variables was observed on multivariate analyses, suggesting a blunting of the relationship due to the effect of other modulators not corrected for on univariate analysis. None of the factors found to be associated with adiponectin accounted for ethnic-group differences in total or HMW adiponectin. Finally, we did not observe the expected negative association between waist circumference and adiponectin in Latino men, suggesting differential regulation of adiponectin in this group.

Our findings of decreased adiponectin in Latinos are similar to those from studies of adiponectin in other minority ethnic/racial groups, where adiponectin is lower in minority groups studied compared to white populations [5,26-28]. Ours is one of the first studies to explore the relationship between adiponectin and several potential regulators of adiponectin in Latinos. Though obesity is known to be associated with decreased adiponectin, differences in adiposity did not explain decreased adiponectin in the Latino group in our study. Furthermore, none of the other potential regulators tested accounted for the observed ethnic differences in adiponectin. Therefore, we conclude that decreased adiponectin in Latinos is determined by genetic or environmental factors we were not able to measure.

Studies on the contribution of adiponectin to differences in insulin sensitivity in Latinos versus non-Latino whites are still lacking. Hanley *et al *reported lower adiponectin levels in African Americans compared to Latinos [31] but comparisons between Latinos and non-Latino whites were not made in that study. Similar to our observations in Latinos versus non-Latino whites, the ethnic difference in adiponectin levels between the African American and Latino groups was no longer significant when insulin sensitivity was taken into account, demonstrating a very close association between these two variables. Contrary to this report, Latasha et al reported lower adiponectin levels in African American women compared to white women with similar insulin sensitivities [32]. Ethnic and racial minority groups (including Latinos) participating in the Diabetes Prevention Program (DPP) study were reported to have lower baseline adiponectin levels than non-Latino white participants [5]. Though adiponectin levels were negatively associated with HOMA-IR in the DPP population as a whole, the association of these two variables was not measured within each racial/ethnic group, and the contribution of decreased adiponectin levels to racial/ethnic differences in insulin sensitivity was not assessed. Our analyses demonstrate that the variability in adiponectin levels in Latinos versus non-Latino whites accounts for the differences in insulin resistance observed in the two groups.

Indo-Asian women have been found to have lower HMW/total adiponectin ratios than whites, suggesting an ethnic-specific regulation of adiponectin isoforms [33]. Contrary to this report however, we found HMW adiponectin to be decreased to a similar extent as total adiponectin in Latinos compared to non-Latino whites. In addition, HMW/total adiponectin ratios did not differ between the two ethnic groups. This observation suggests the involvement of mechanisms regulating adiponectin production, with sparing of mechanisms involved in adiponectin multimerization. Consistent with observations in majority populations [34], HMW adiponectin was more strongly associated with insulin sensitivity than total adiponectin in our population. However, both total and HMW adiponectin levels were associated with insulin sensitivity, and the ethnic-specific difference in insulin sensitivity was no longer apparent when either adiponectin measure was controlled for in our regression models. To our knowledge, ours is the first study to compare HMW adiponectin in Latinos and non-Latino whites and to identify determinants of HMW adiponectin in Latinos.

While we are not able to establish a causative association between adiponectin and insulin action in this cross-sectional study, multiple studies have shown that adiponectin has insulin-sensitizing actions (reviewed in [35]). In addition, multiple prospective studies and a recent meta-analysis have demonstrated that individuals with low adiponectin are at higher risk for development of type 2 diabetes [5,36]. In the DPP study [5] baseline adiponectin was a strong independent predictor of incident diabetes in the three treatment groups (lifestyle, metformin, and placebo), with a hazard ratios of 0.61-0.79 per 3 microgram/ml higher adiponectin). Thus, our findings suggest that decreased adiponectin in Latinos plays a role in the high incidence of insulin resistance and type 2 diabetes in this population. As in other populations, the HMW form of adiponectin is a better determinant of insulin sensitivity than total adiponectin. However, the observed decrease in adiponectin in the Latino population is not isoform-specific.

We found an expected negative association between waist circumference and adiponectin in women from both ethnic groups, and in the non-Latino white men, but no correlation between these two variables in the Latino men group. Though we used waist circumference as a surrogate measure of visceral adiposity, our findings are consistent with those of Hanley *et al *who reported an inverse correlation between visceral adiposity and adiponectin in African Americans but not Mexican Americans [31]. These findings may indicate ethnic differences in adiponectin regulation. For instance, it is possible that Latino men are more sensitive to inhibitory visceral cytokines, resulting in an early plateau of suppressed adiponectin levels at a lower waist circumference. Alternatively, other (non-visceral) inhibitory factors may have a stronger influence on adiponectin levels in Latinos.

Our study has several limitations. Our population consisted of mostly older, overweight or obese adults, who had a diagnosis of hypertension and one additional risk factor for CVD. Therefore, our findings may not apply to a younger, leaner, or healthier population. Also, our Latino group was largely comprised of Mexican Americans, and thus our results may not be generalizable to other Latino groups. As this was a cross-sectional study, we cannot determine directionality in the correlations observed or fully explore the causes of low adiponectin in Latinos. Additional limitations of the study include our use of the homeostasis model, a surrogate marker of insulin sensitivity, rather than a more direct measure such as a frequently sampled IV glucose tolerance test or a hyperinsulinemic euglycemic clamp. Finally, we used waist circumference as an estimation of visceral adiposity rather than a more direct measurement such as can be obtained with CT scans.

## Conclusions

We report decreased adiponectin levels in Latinos at increased risk of cardiovascular disease, when compared to non-Latino whites, independent of adiposity measures. While our study confirms previously described associations between adiponectin and age, gender, thiazolidinedione use, and adiposity, none of these variables accounted for the observed ethnic differences in adiponectin levels. Indeed, after controlling for other variables correlated to adiponectin, Latino ethnicity remained the strongest negative predictor of both total and HMW adiponectin levels. In addition, our findings suggest differential regulation of adiponectin in these two ethnic populations, with a negative association between waist circumference and adiponectin observed in women and non-Latino white men but not in Latino men. The ethnic-specific differences we have observed could be a reflection of differences in genetics or in environmental factors such as diet. Decreased adiponectin in Latinos may contribute to increased insulin resistance and thereby increased incidence of type 2 diabetes in this population. These findings suggest that therapeutic interventions that increase adiponectin levels may have a beneficial impact on the prevention of type 2 diabetes in Latinos. Future studies focusing on the mechanisms for decreased adiponectin in Latinos and the metabolic consequences of ethnic differences in adiponectin levels seem warranted.

## List of abbreviations

ACEI: Angiotensin-converting enzyme inhibitors; ARB: angiotensin receptor blockers; BMI: body mass index; CT: computed tomography; CVD: Cardiovascular disease; eGFR: estimated glomerular filtration rate; ELISA: Enzyme-linked immunosorbent assay; hsCRP: high sensitivity C-reactive protein; HOMA-IR: Homeostasis model assessment insulin resistance index; HMW: High-molecular-weight adiponectin isoform; MDRD: Modification of Diet in Renal Disease; RIA: Radioimmunoassay; T2DM: Type 2 diabetes mellitus; TZD: thiazolidinediones.

## Competing interests

The authors declare that they have no competing interests.

## Authors' contributions

RIP conceived of the study, participated in the design, supervised the measurements of HMW adiponectin, and drafted the manuscript. PH performed the statistical analyses and was involved in the interpretation of the data. LMD contributed to the study design, analyses, and interpretation. CCLW, MC, and MLK were involved in the analyses, interpretation of the data and critical review of the manuscript. JFS supervised all aspects of the LUCHAR study, contributed to the interpretation of the data, and critically reviewed the manuscript. DHB contributed to the interpretation of the data and critically reviewed the manuscript. CSL and EPH conceived of the CVD part of the LUCHAR study, contributed to the study design and data interpretation for the present study, and critical reviewed the manuscript. All authors read and approved the final manuscript.

## Authors' information

RIP is a Staff Endocrinologist in the Community Health Services at Denver Health and an Assistant Professor at the University of Colorado Anschutz Medical Campus, Division of Endocrinology, Diabetes, and Metabolism.

## Pre-publication history

The pre-publication history for this paper can be accessed here:

http://www.biomedcentral.com/1472-6823/11/13/prepub

## Supplementary Material

Additional file 1 **Figure S1 - Relationship between total adiponectin level and waist circumference in Latino and non-Latino white men and women**. Scatter-plot and predicted regression lines showing the relationship between total adiponectin and waist circumference by ethnicity and gender. Data for Latino women is represented by *blue symbols *and *blue line; *for Latino men by *green symbols *and *green line*, non-Latino white women by *red symbols *and *red line*; and for non-Latino white men by *brown symbols *and *brown line*. Click here for file

Additional file 2 **Figure S2 - Relationship between total adiponectin level and BMI in Latino and non-Latino white men and women**. Scatter-plot and predicted regression lines showing the relationship between total adiponectin and BMI by ethnicity and gender. Data for Latino women is represented by *blue symbols *and *blue line; *for Latino men by *green symbols *and *green line*, non-Latino white women by *red symbols *and *red line*; and for non-Latino white men by *brown symbols *and *brown line*. Click here for file

Additional file 3 **Table S1 - Linear regression analysis: Association between adiponectin and clinical characteristics**. Table S2 - Multivariate linear regression analysis: Association between logHOMA-IR and BMI/waist circumference, ethnicity, and adiponectin. Click here for file
