# Supplementary figures and images for: Circulating adiponectin levels are lower in Latino versus non-Latino white patients at risk for cardiovascular disease, independent of adiposity measures
Source: BMC Endocr Disord. 2011 Jul 7;11:13. doi: 10.1186/1472-6823-11-13 (PMC3141565; doi:10.1186/1472-6823-11-13)

## Slide 1
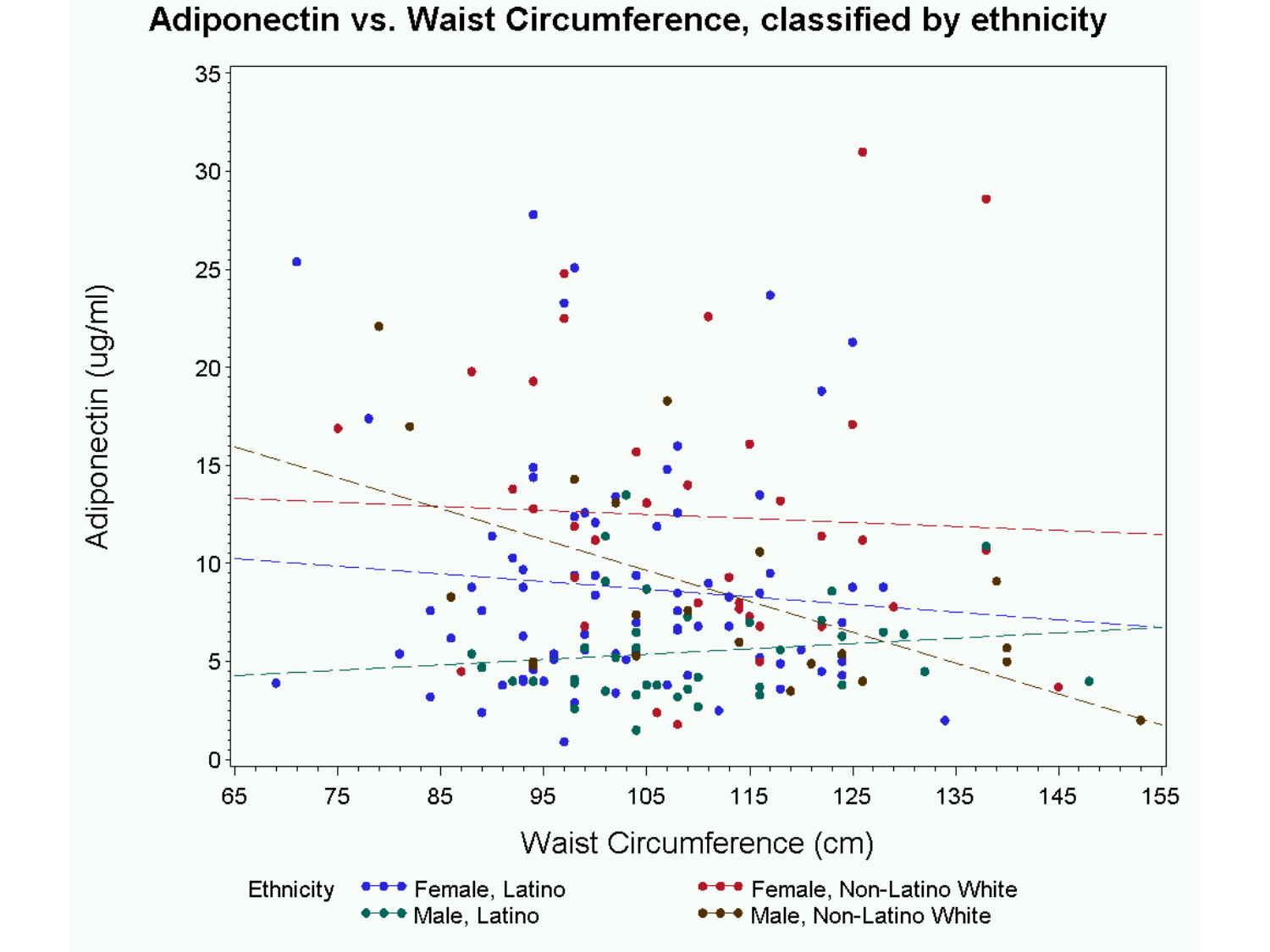

Supplement: Additional file 1 — Figure S1 - Relationship between total adiponectin level and waist circumference in Latino and non-Latino white men and women. Scatter-plot and predicted regression lines showing the relationship between total adiponectin and waist circumference by ethnicity and gender. Data for Latino women is represented by blue symbols and blue line; for Latino men by green symbols and green line, non-Latino white women by red symbols and red line; and for non-Latino white men by brown symbols and brown line. [file 1472-6823-11-13-S1.PPT]

## Slide 1
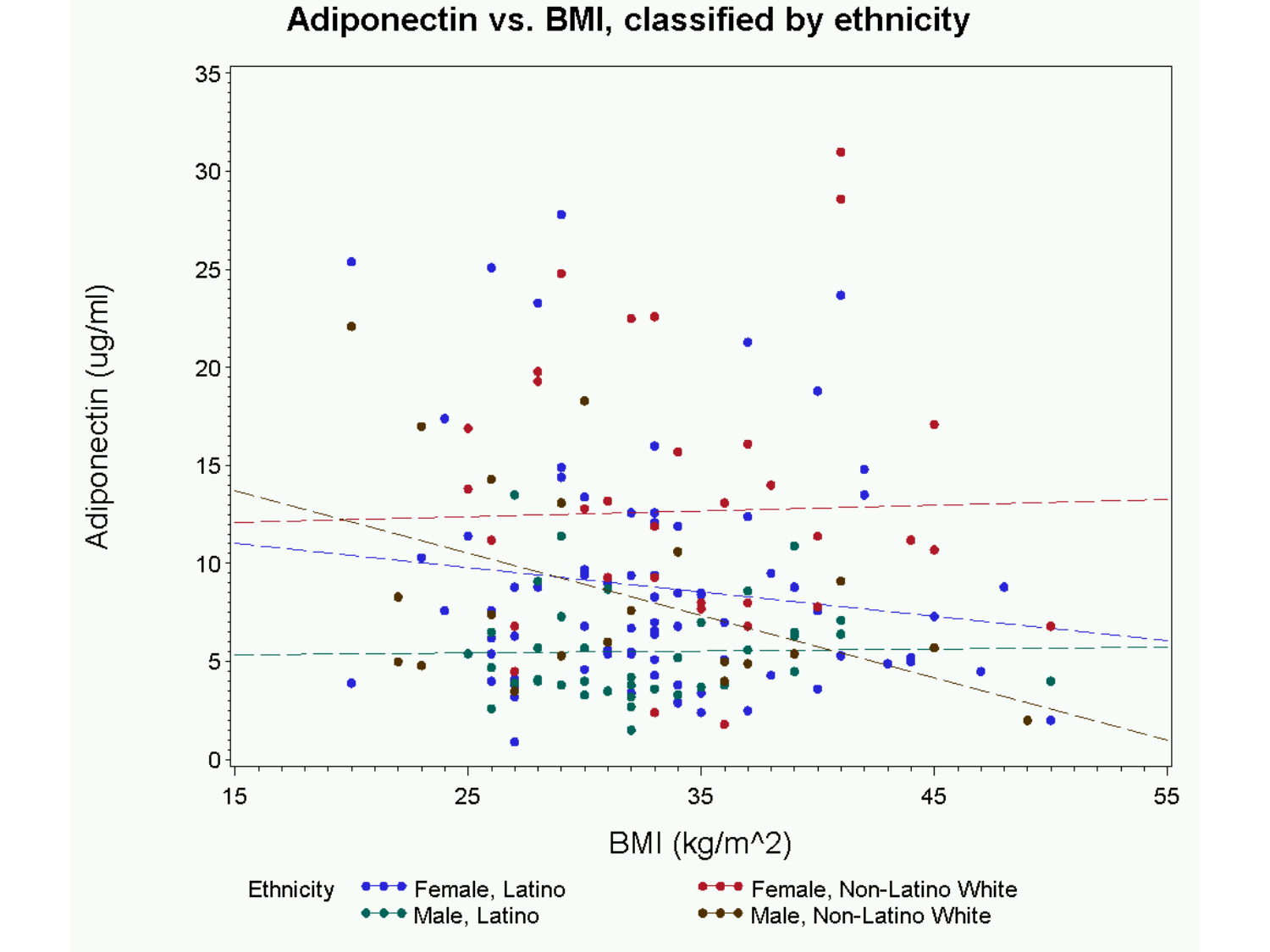

Supplement: Additional file 2 — Figure S2 - Relationship between total adiponectin level and BMI in Latino and non-Latino white men and women. Scatter-plot and predicted regression lines showing the relationship between total adiponectin and BMI by ethnicity and gender. Data for Latino women is represented by blue symbols and blue line; for Latino men by green symbols and green line, non-Latino white women by red symbols and red line; and for non-Latino white men by brown symbols and brown line. [file 1472-6823-11-13-S2.PPT]
